# Superimposed Whole-Body Electrostimulation Augments Strength Adaptations and Type II Myofiber Growth in Soccer Players During a Competitive Season

**DOI:** 10.3389/fphys.2019.01187

**Published:** 2019-09-23

**Authors:** Andre Filipovic, Markus DeMarees, Marijke Grau, Anna Hollinger, Benedikt Seeger, Thorsten Schiffer, Wilhelm Bloch, Sebastian Gehlert

**Affiliations:** ^1^Section of Molecular and Cellular Sport Medicine, Institute of Cardiology and Sports Medicine, German Sport University Cologne, Cologne, Germany; ^2^Section of Sports Medicine and Sports Nutrition, Faculty of Sports Science, Ruhr-University Bochum, Bochum, Germany; ^3^Outpatient Clinic for Sports Traumatology and Public Health Consultation, German Sport University Cologne, Cologne, Germany; ^4^Institute of Sport Science, Biosciences of Sports, University of Hildesheim, Hildesheim, Germany

**Keywords:** electrostimulation, soccer, hypertrophy, mTOR, p70s6k, strength

## Abstract

**Background:**

The improvement of strength and athletic performance during a competitive season in elite soccer players is a demanding task for the coach.

**Aims:**

As whole-body electrostimulation (WB-EMS) training provides a time efficient stimulation potentially capable in exerting skeletal muscle adaptations we aimed to test this approach over 7 weeks in trained male soccer players during a competitive season.

**Hypothesis:**

We hypothesized that a superimposed WB-EMS will increase maximal strength and type I and type II myofiber hypertrophy.

**Methods:**

Twenty-eight male field soccer players were assigned in either a WB-EMS group (EG, *n* = 10), a training group (TG, *n* = 10), or a control group (CG, *n* = 8). The regular soccer training consists of two to four sessions and one match per week. In concurrent, the EG performed 3 × 10 squat jumps superimposed with WB-EMS twice per week, TG performed 3 × 10 squat jumps without EMS twice per week, and the CG only performed the regular soccer training. Muscle biopsies were collected and strength tests were performed under resting conditions before (Baseline) and after the intervention period (Posttest). Muscle biopsies were analyzed via western blotting and immunohistochemistry for skeletal muscle adaptive responses. To determine the effect of the training interventions a 2 × 3 (time ^∗^ group) mixed ANOVA with repeated measures was conducted.

**Results:**

Maximal strength in leg press (*p* = 0.009) and leg curl (*p* = 0.026) was significantly increased in EG along with a small but significant increase in type II myofiber diameter (*p* = 0.023). All of these adaptations were not observed in TG and CG.

**Conclusion:**

WB-EMS can serve as a time efficient training method to augment strength capacities and type II fiber myofiber growth in soccer players when combined with specific resistance training. This combination may therefore be a promising training modification compared to traditional strength training for performance enhancement.

## Introduction

The improvement of physical performance of soccer players in short time courses is of crucial importance in high performance soccer. Here, running distances associated with high intensity have significantly increased in the last decade (Mohr et al., 2003; Di Salvo et al., 2010). Consequently physical requirements, especially the development of strength capacities, gained more importance. But with increasing number of games per season, a well-developed physical robustness and muscular performance also plays a crucial role in the context of injury prevention (Al Attar et al., 2017). However, assuring adequate recovery after a match and timelike integrating effective strength programs to systematically increase soccer-relevant strength parameters during the competitive season is a demanding task for the coach. Due to a lack of time, the implementation of alternative training methods that offer high efficient stimulation of muscle adaptation has increasing value. Among these, stimulation of muscle via electromyostimulation (EMS) to increase maximal strength and specific strength capacities such as jumping and sprinting of trained and elite team sport athletes seems promising (Delitto et al., 1989; Maffiuletti et al., 2000, 2002a,b; Malatesta et al., 2003; Gondin et al., 2005; Billot et al., 2010; Filipovic et al., 2016). The electrical stimulus leads to a mostly indirect involuntary contraction controlled by the central nervous system (Gregory and Bickel, 2005). Studies revealed that low frequency (approx. 50–120 Hz) EMS can produce a high muscle tension and thus a high metabolic and mechanical stress on the muscular structures (Gregory and Bickel, 2005; Jubeau et al., 2008; Nosaka et al., 2011) that trigger neuronal and hormonal adaptation processes (Paillard, 2008).

Studies showed that a single bout of intense whole-body electrostimulation (WB-EMS) can produce a high level of muscular damage or even rhabdomyolysis especially when it is performed with isometric contractions and with maximal intensity (Kemmler et al., 2015; Stollberger and Finsterer, 2019). Adusting the training intensity under EMS is difficult. Thus, when not applied with care, EMS exercises may induce health risks and bring this training mode under discussion. Thus, in the last years, studies were conducted to investigate the effects on health parameters and performance (Jee, 2018; Kemmler et al., 2018), and guidelines were developed for a safe and efficient implementation (Kemmler et al., 2016). But it is generally accepted, that when properly applied and supervised, WB-EMS represents a safe training method in healthy adults to develop physical performance and health.

Several authors conclude that the increases in maximal strength are mainly through a synchronous activation and higher frequency of motor units, as well as preferential recruitment of fast twitch fibers with relatively low muscle activity (Martin et al., 1994; Pichon et al., 1995; Colson et al., 2000; Maffiuletti et al., 2000, 2002b; Dudley and Stevenson, 2003). However, studies indicate that EMS can activate muscular adaptations and affect muscle characteristics such as a muscle fiber shift toward type IIa fibers (Cabric and Appell, 1987; Delitto et al., 1989; Bigard et al., 1993; Perez et al., 2002; Requena Sanchez et al., 2005) and can promote muscle hypertrophy (Gondin et al., 2005, 2011; Maffiuletti et al., 2006). Hortobagyi and Maffiuletti (2011) concluded in their review that an increase in maximum voluntary contraction in the early stage may not be due to muscle hypertrophy, but rather through changes within structures of the central nervous system. However, hypertrophic effects are possible with EMS, whereas these may only occur during longer stimulation periods of >6 weeks.

One of the major signaling pathways that regulates increased protein synthesis and muscle hypertrophy is the PI3kinase/Akt/mTOR pathway (Egerman and Glass, 2014). Even though limited data are available, some EMS studies have shown that EMS can stimulate the release of growth hormones or insulin-like-growth factor-1 (IGF-1) that can activate the mTOR-signaling pathway (Jubeau et al., 2008; Gondin et al., 2011; Wirtz et al., 2015). However, it has been shown that exercise-induced protein synthesis and muscle hypertrophy can be activated also in the absence of a release of growth hormones and IGF-1 (cf. Wilkinson et al., 2006; Schroeder et al., 2013; Egerman and Glass, 2014). Studies revealed that the mechanical strain of skeletal muscles can directly stimulate mTOR-related signaling (Jacobs et al., 2014) and p70s6k phosphorylation (Sandilands et al., 2015) via focal adhesion kinase. P70s6k has been shown to correlate with muscle growth and protein synthesis (Terzis et al., 2008, 2010). As EMS induces a significant mechanical stimulation of skeletal muscle fibers, assessment of p70s6k levels after EMS stimulation is promising.

In our previous study with professional soccer players (Filipovic et al., 2016) we achieved significant increases in maximal strength of the leg press (LP), jumping and sprinting performance with a dynamic WB-EMS training after 7 weeks (14 sessions). In this study we were not able to investigate skeletal muscle substructures; thus, it remains unclear whether muscle hypertrophy has occurred and an increase in muscle size has positively influenced maximal strength. Although the effects of EMS in various setups have been investigated, there is still a lack of knowledge concerning its application, e.g., in high performance soccer players, as a time-efficient training enhancement within a season and regular soccer training (cf. Billot et al., 2010; Filipovic et al., 2012, 2016).

Based on the capacity of EMS to significantly affect the neuromuscular level of skeletal muscle, we aimed to test in the present study whether EMS-induced strength gains are also associated with molecular and structural adaptations in skeletal muscle of high performance soccer players, when WB-EMS is applied with specific strength training during a continuous soccer training regimen. We hypothesized that WB-EMS in combination with a specific jumping training will increase strength capacities in skeletal muscle but not in non-WB-EMS stimulated athletes. We further hypothesized that this response was associated with increased diameter of myofibers reflected by increased protein levels of p70s6k and mTOR.

## Materials and Methods

### Participants

Only healthy field soccer players were included which means no cardiovascular or metabolic diseases and no preinjury in the tested muscle groups. Participants needed to compete on a regional level for the last 3 years and train two to four sessions per week and play one soccer match per week. Experience in strength training was required. Twenty-eight soccer players were randomly assigned into three different groups. Control group (CG) was assigned based on preferences and availability, whereas both intervention arms have been assigned based on coin toss. The EMS group (EG, *n* = 10) performed 3 × 10 squats jumps superimposed by WB-EMS twice a week in addition to the regular soccer routine over a period of 7 weeks. To differentiate between the effects caused by EMS and by the squat jumps and soccer training, respectively, two CGs were included. A jump training group (TG, *n* = 10) performed the same number of squat jumps with identical intervals without EMS stimulus on the same days as the EG and a CG (*n* = 8) that only performed the regular soccer routine.

Basal anthropometric parameters of the subjects are shown in Table 1. All subjects abstained from alcohol consumption for 24 h prior to the Baseline diagnostics and during the training intervention and were non-smokers.

**TABLE 1:** Anthropometric data (mean ± *SD*) and total training load (arbitrary units) during the 7-week intervention period calculated by Polar Team-2 Software according to training time spent in defined heart rates (see the section “Materials and Methods”).

**Group**	**Age (year)**	**Height (m)**	**Weight (kg)**	**Bodyfat (%)**	**VO_2_peak**	**Sessions/week**	**Total training load (Polar Team-2)**
EG	24.4 ± 4.2	1.82 ± 0.03	81.4 ± 5.3	12.9 ± 2.1	52.1 ± 3.4	3.4 ± 1.2	3430.6 ± 910.7
TG	21.1 ± 1.9	1.83 ± 0.06	79.7 ± 5.5	10.8 ± 2.8	56.3 ± 5.7	3.4 ± 1.3	3478.6 ± 1722.8
CG	23.6 ± 3.9	1.82 ± 0.05	79.7 ± 7.5	14.1 ± 3.6	54.3 ± 7.2	2.6 ± 0.7	2644.4 ± 1437.3

Twenty-seven players completed the two strength diagnostics. One player of the TG dropped out from the study because of an ankle joint injury before the Posttest and one sample could not be analyzed due to a missing Posttest of one subject. This subject was not willing to conduct a second biopsy. Muscle samples from 25 subjects were used for Western blotting and histology.

### Definition of Daily Soccer Routine

The regular soccer training consists of 3.2 ± 1.0 sessions per week with a soccer match at the end of the week (90 min). The standard training sessions lasted 80.7 ± 10.1 min including general and specific warm-up (light to moderate intensity), athletic components with various intensities, technical skill activities (light to moderated intensity), offensive and defensive tactics (light to moderate intensity), small-sided game plays (e.g., 4 vs. 4 – 32 × 40 m; high intensity) and continuous play (e.g., 8 vs. 8 – 60 × 60 m; 10 vs. 10 – 100 × 60 m; moderate to high intensity). In a normal training week during season with a match on Sunday training was scheduled on Tuesdays, Wednesdays (optional), Thursdays, and Fridays. Number of training sessions and the training days varied according to the game schedule playing Sunday–Sunday or Sunday–Saturday. The number of training sessions and the total training minutes were documented. Training load included matches and was measured via Polar Team-2 Software (Polar Electro, Büttelborn, Germany) according to the training time spent in defined heart rate zones and related to the individual maximum heart rate during soccer training or match (Table 1). The training load provided by the Polar-Software determines the internal training load based on background variables [sex, training history, metabolic thresholds, and maximal oxygen consumption (VO_2_max)] and parameters measured during training sessions (exercise mode, heart rate, and energy expenditure) (cf. Schumann et al., 2017). The individual maximum heart rate and maximum oxygen uptake VO_2_peak of the players were measured in a maximal ramp test for calculation of training load via the Polar-software.

The players were asked to maintain their usual food intake and hydration and no nutrition supplementation was used. Additional strength training was not allowed during the study.

All players had a constant training volume during the first half of the season (July till December) and were in a well-trained condition. The intervention period started after the 3 week mid-season break from end of December till mid of January. During these 3 weeks the training load was relatively low (moderate endurance training twice per week) in order to maintain fitness level and not negatively affect baseline testing.

### Exercise Protocol

Whole-body electrostimulation training was conducted on Tuesdays and Fridays in order to obtain a rest interval of 48 h between the two sessions and the championship game on Sunday. The EMS training was conducted using a WB-EMS-system by “*miha bodytec*” (Augsburg, Germany). WB-EMS was applied with an electrode vest to the upper body with integrated bilaterally two paired surface electrodes for the chest (10 × 4 cm), upper and lower back (14 × 11 cm), latissimus (10 × 4 cm), and the abdominals (23 × 10 cm) and with a belt system to the lower body including the muscles of the glutes (13 × 10 cm), thighs (44 × 4 cm), and calves (27 × 4 cm). Biphasic rectangular wave pulsed currents (80 Hz) were used with an impulse width of 350 μs (cf. Filipovic et al., 2016). The stimulation intensity (0–120 mA) was determined and set separately for each muscle group by using a Borg Rating of Perceived Exertion (cf. Tiggemann et al., 2010). The training intensity was defined for each players in a familiarization session 2 weeks before and set at a sub-maximal level that still assures a clean dynamic jump movement (RPE 16–19 “hard to very hard”) and was saved on a personalized chip card. The EG performed 3 × 10 maximal squat jumps with a set pause of 60 s (no currents) per session. Every impulse for a single jump lasted for 4 s (range of motion: 2 s eccentric from standing position to an knee angle of 90° – 1 s isometric – 0.1 s explosive concentric – 1 s landing and stabilization) followed by a rest period of 10 s (duty cycle approx. 28%). The total duration time was 8.5 min per session with an effective stimulation time of 2 min per session. The players started with a 2–3 min standardized warm-up with movement preparations including squats, skipping, and jumps in different variations (squat jumps, jumps out of skipping, or double jumps) at a light to moderate stimulation intensity. The players were told to slowly increase the intensity every few impulses. The training started when the players reached the defined training intensity that was saved on the chip card from the last session according to the RPE 16–19 (“hard to very hard”). The stimulation intensity was constantly increased individually every week (Tuesdays) in order to maintain a high stimulation intensity.

The TG performed the same amount of jumps with identical interval and conduction twice per week without EMS. The CG only performed the two to four soccer training session plus one match per week.

### Experimental Protocol

#### Strength Diagnostics

Isometric strength and isoinertial power diagnostics were performed with the LP and leg curl (LC) machine (Edition-Line, gym80, Gelsenkirchen, Germany) equipped with digital measurement technique Digimax (mechaTronic, Hamm, Germany) and according to the protocol of the Institute of Training Science and Sport Informatics at the German Sport University Cologne (cf. Wirtz et al., 2016). The force-time and velocity-time variables were measured via strength sensors (5 kN strength sensor typ KM1506, distance sensor typ S501D, megaTron; Munich, Germany) and analyzed with the softwares IsoTest and DynamicTest 2.0. The sensors were installed in line with the steel band of the machines that lifts the selected weight plates. The maximum force in relation to body weight [Frel (N⋅kg^–1^)] was calculated and used for statistical analysis. After a 10 min standardized warmup including cycling on a ergometer and one set of 10 reps with moderate weight (approx. 40% 1RM) at the LP and the LC machine the players performed three isometric tests per test machine to measure maximal strength. Isometric attempts were conducted at an inner knee angle of 120°.

#### Muscle Biopsies and Tissue Treatment

**FIGURE 1:**
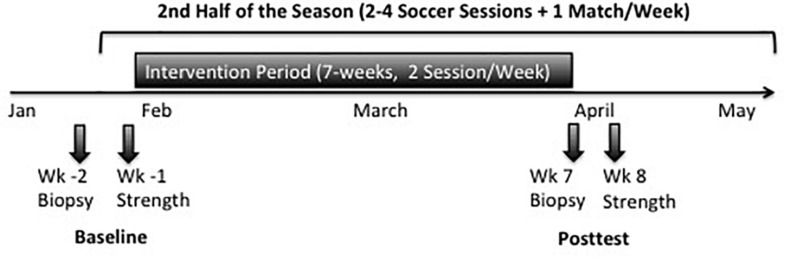
Timeline of strength testing and muscle biopsies during the study in the second half of the season.

Muscle biopsies were taken via Bergström needle biopsies (Bergstrom, 1975) from each player 2 weeks before (Baseline) and 48 h after the last training intervention (Posttest) (Figure 1). All biopsies were obtained under local anesthesia from the middle portion of the *m. vastus lateralis* between the lateral part of the patella and spina iliaca anterior superior 2.5 cm below the fascia. The muscle samples were freed from blood and non-muscular material and embedded in tissue freezing medium (TISSUE TEK, Sakura, Zoeterwoude/Netherlands). Samples were frozen in liquid nitrogen-cooled isopentane and stored at −80°C for further analysis. The distance between Baseline and Posttest incision was approx. 2.5 cm.

#### Immunohistochemistry

Seven micrometers of cross-sectional slices were obtained from the frozen muscle tissue using a cryo-microtome Leica CM 3050 S (Leica Microsystems, Nußbach, Germany) and placed on Polysine^TM^ microscope slides (VWR International, Leuven, Belgium). Sections were fixed for 8 min in −20°C pre-cooled acetone and air dried for 15 min at room temperature (RT), before blocking for 1 h at RT with Tris-buffered saline (TBS, 150 mM NaCl, 10 mM Tris–HCl, pH 7.6) containing 5% bovine serum albumin (BSA). After blocking, sections were incubated over night with rabbit polyclonal primary antibody which detects type I Myosin Heavy Chain (host: mouse; A4.951, DSHB Iowa, IA, United States), diluted 1:200 in 0.8% BSA. To confirm antibody specificity, control sections were incubated in TBS containing 0.8% BSA but without primary antibody. After incubation, sections were washed three times with TBS and incubated for 1 h with biotinylated goat anti-mouse secondary antibody (Dako, Glostrup, Denmark), diluted 1:400 in TBS, at RT. Thereafter, sections were washed three times before incubation for 1 h at RT with streptavidin biotinylated horseradish peroxidase complex (Amersham Biosciences, Uppsala, Sweden) 1:400 in TBS. Sections were washed once again and immunohistochemical staining was finalized using a 3,3′-diaminobenzidine (DAB) solution (0.09 M phosphate buffer, pH 7.4; 2.2 mM DAB; 7.03 mM ammonium chloride; 0.93 mM nickel sulfate; 10.44 mM β-D-glucose and 0.024 μM glucose oxidase). The procedures described are in accordance with the standard protocol for immunohistochemistry of the Institute of Molecular and Cellular Sport Medicine at the German Sport University Cologne (Germany) and have been described in detail before by Jacko et al. (2018).

#### Westernblotting

**FIGURE 2:**
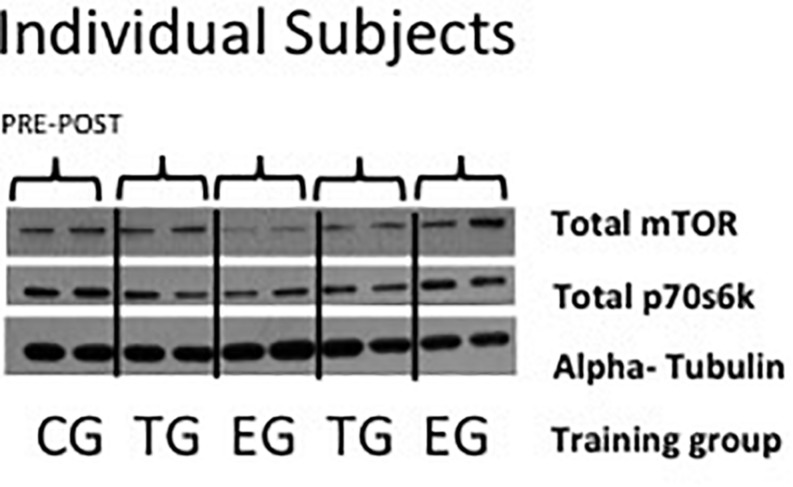
Representative picture of Western blots of individual subjects of total mTor, total p70s6k, and alpha-tubulin in EMS-Group (EG), Training-Group (TG), and Control-Group (CG) measured 2 weeks before (Pre) and after the intervention period (Post).

Tissue was homogenized in ice-cold lysis buffer (Cell Signaling, Boston, MA, United States) using a micro-dismembrator (Braun, Melsungen, Germany). Homogenates were supplemented with a mixture of protease and phosphatase inhibitors (HaltTM, Thermo Scientific, Waltham, MA, United States). The protein concentration of each sample was quantified with a Lowry test kit (Bio Rad, Munich, Germany) on a multiplate reader (Multiscan FC, Thermo Scientific, Waltham, MA, United States). For gel electrophoresis, 3× Laemmli buffer was added to the samples and heated at 95°C for 5 min. Afterward samples were stored on ice but brought to RT before being loaded on a precast 6–12% *bis*–*tris* polyacrylamide gel system (Criterion^TM^ XT, Bio Rad, Munich, Germany). After electrophoretic separation (100 volt, constant) in XT MOPS Running Buffer (Bio Rad, Munich, Germany), proteins were transferred (1.2 amp, 25 volt max, 34 min) to a polyvinylidene difluoride (PVDF) membrane (Bio Rad, Munich, Germany) using semi dry blotting systems (Criterion^TM^, Bio-Rad, Munich, Germany). Membranes containing the separated proteins were blocked in 5% non-fat dry milk for 1 h at RT and incubated over night at 4°C with total mTOR (NEB 2983) and total p70s6k (NEB 2708) specific antibodies (Cell Signaling, Boston, MA, United States). Goat anti-rabbit antibodies diluted 1:1500 in 5% BSA, membranes were washed three times with TBST [TBS added with 1% Tween120 (Sigma–Aldrich, St. Louis, MO, United States)] and afterward incubated for 1 h at RT with the secondary antibody (goat anti-rabbit, diluted 1:10.000 in 5% non-fat dry milk, Thermo Scientific, Rockford, United States) and then washed in TBST. Proteins were detected by an enhanced chemo-luminescence assay (ECL-Kit, Amersham Life Science, Buckinghamshire, United Kingdom) exposed to an X-ray film (Kodak, X-OMAT Engineering, Eastman Kodak Co., Rochester, NY, United States) (Figure 2).

#### Determination of Myofiber Diameter

**FIGURE 3:**
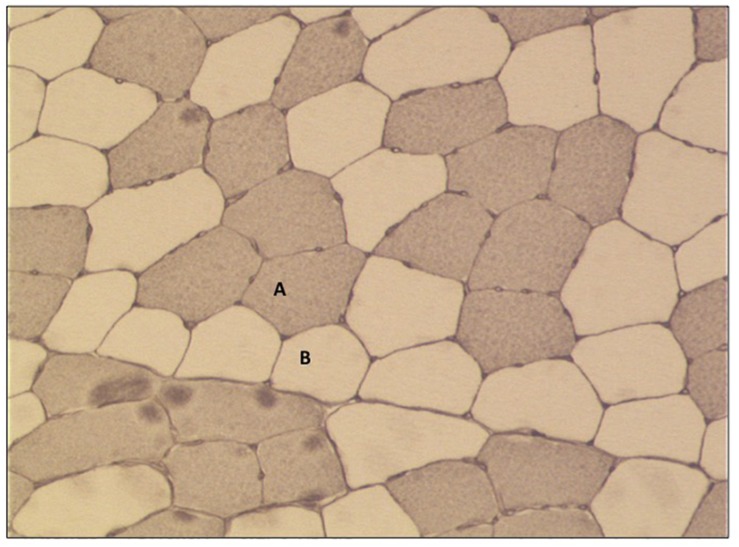
Representative picture of immunohistochemical staining of myofiber cross-sections (10× fold magnification). **(A)** Type I fibers (stained) and **(B)** type II fibers (no stain).

All slides were examined with a Zeiss Axiophot 200 light microscope coupled to a Sony 3CCD Color Video Camera. Up to 10 digital pictures were taken from each cross section with 10-fold magnification (10-fold objective) (Figure 3). A digital micrometer scale was applied to set the correct μm/pixel ratio for this magnification level. 1.2 pixel determined a length of 1 μm and this aspect ratio was used for all photos. Myofiber diameter was quantified by selection of the sarcoplasmic region within the borders of the sarcolemma of the myofiber and its subsequent measurement by the best fitting ellipse tool (Cluff et al., 2013) using the software ImageJ^®^ (National Institutes of Health, United States). The minor axis was determined as the diameter of the myofiber. 55.4 ± 8.2 type I and type II myofibers per subject and time point analyses for changes in myofiber diameter.

### Statistical Analysis

All descriptive and inferential statistical analyses were conducted using SPSS 25^®^ (IBM^®^, Armonk, NY, United States). Kolmogorov–Smirnov test was applied to test for normal distribution. Data for muscle fiber size and mTOR were normally distributed. To determine the effect of the training interventions on maximal strength, muscle fiber size, and the signal protein, a separate 2 × 3 (time ^∗^ group) mixed ANOVA with repeated measures was conducted. ANOVA assumption of homogenous variances was tested using Mauchly’s test of Sphericity. Greenhouse–Geisser correction was used when a violation of Mauchly’s test was observed. Partial eta-square (${\text{η}}_{\text{p}}^{2}$) values are reported as effect size estimates. If 2 × 3 mixed ANOVA revealed a significant time-point ^∗^ treatment or time ^∗^ group interaction effect on any variable, this effect was further investigated carrying out Bonferroni corrected *post hoc* pairwise comparison. Group differences were determined by a one-way ANOVA. Bonferroni *post hoc* test was used to calculate significant differences between the tested groups. Effect size Cohen’s *d*, defined as difference in means/standard deviation was calculated for groups between Baseline and Posttest (0.2–0.4 small; 0.5–0.7 medium; >0.8 large effects) (Cohen, 1988).

To detect correlations between the increase in Frel of LP and LC and muscle fiber size Pearson product-moment correlation (one-tailed) was used.

## Results

Changes of anthropometric data and the results of maximal strength testing of the players are shown in Table 2. The results in type I and type II myofiber diameter are presented in Table 3.

**TABLE 2:** Results for anthropometric data and relative maximal strength (Frel) at leg press and leg curl for EMS-Group (EG), Training-Group (TG), and Control-Group (CG) 1 week before (Baseline) and 1 week after intervention period (Posttest).

	**Group**	***n***	**Baseline (MW ± *SD*)**	**Posttest (MW ± *SD*)**	**Delta (baseline vs. Posttest) (%)**	***Post hoc* Bonferroni correction (*p*-value)**	**Effect size (Cohen’s *d*)**
Bodyweight (kg)	EG	10	81.4 ± 5.3	81.7 ± 5.4	0.3 ± 2.8	0.741	0.11
	TG	10	79.2 ± 5.7	79.7 ± 5.5	0.6 ± 2.6	0.243	0.41
	CG	8	80.2 ± 7.3	80.9 ± 6.5	1.0 ± 2.7	0.519	0.25
Bodyfat (%)	EG	10	12.9 ± 2.2	12.5 ± 2.1	−2.3 ± 12.7	0.494	0.24
	TG	10	10.6 ± 2.7	10.3 ± 2.5	−3.0 ± 7.9	0.176	0.01
	CG	8	14.1 ± 3.4	14.0 ± 4.5	−0.9 ± 19.6	0.812	0.49
Leg press Rel. Fmax (N⋅kg^–1^)	EG	10	41.1 ± 9.3	47.0 ± 11.1^∗∗^	15.1 ± 13.3	0.009	1.11
	TG	9	43.3 ± 11.5	46.7 ± 12.4	8.6 ± 15.6	0.203	0.49
	CG	8	41.9 ± 11.4	43.8 ± 16.9	4.3 ± 14.9	0.436	0.31
Leg curl Rel. Fmax (N⋅kg^–1^)	EG	10	17.9 ± 1.5	19.3 ± 2.2^∗^	8.5 ± 9.3	0.026	0.89
	TG	9	17.4 ± 2.4	18.2 ± 1.7	4.7 ± 9.2	0.225	0.47
	CG	8	16.6 ± 2.2	16.4 ± 2.3	2.9 ± 4.7	0.796	0.10

*Values are presented in means ± *SD*, significant differences at ^∗^*P* < 0.05, ^∗∗^*P* < 0.01.*

**TABLE 3:** Results for myofiber diameter (minor axis) in Type I and Type II fibers in EMS-Group (EG), Training-Group (TG), and Control-Group (CG) 2 weeks before (Baseline) and after intervention period (Posttest).

**Muscle fiber type**	**Group**	***n***	**Baseline (minor axis) (MW ± *SD*) (μm)**	**Posttest (minor axis) (MW ± *SD*) (μm)**	**Delta (baseline vs. posttest) (%)**	***Post hoc* Bonferroni correction (*p*-value)**	**Effect size (Cohen’s *d*)**
Type I	EG	8	69.39 ± 5.03	72.18 ± 5.63	4.1 ± 5.5	0.088	0.75
	TG	9	71.83 ± 5.10	72.90 ± 4.40	1.8 ± 7.9	0.599	0.19
	CG	8	72.80 ± 5.60	75.00 ± 5.96	3.3 ± 6.9	0.279	0.44
Type II	EG	8	74.10 ± 6.14	80.36 ± 5.53^∗^	8.9 ± 8.5	0.023	1.10
	TG	9	76.56 ± 5.78	78.35 ± 7.38	2.6 ± 10.0	0.505	0.24
	CG	8	77.75 ± 7.67	79.23 ± 5.08	2.7 ± 10.5	0.629	0.19

*Values are presented in means ± *SD*, significant differences at ^∗^*P* < 0.05, ^∗∗^*P* < 0.01.*

### Anthropometric Data and Training Load

No changes were observed in bodyweight (kg) or bodyfat (%) from Baseline to Posttest. CG tends to have a higher percentage of bodyfat in general but no group differences could be observed between the groups at Baseline or Posttest.

Regarding training volume and load no differences were observed between the groups in the total number of soccer training sessions (EG 23.9 ± 7.8; TG 25.9 ± 6.6; CG 18.1 ± 5.6 sessions), soccer training minutes (EG 2103 ± 630; TG 1812 ± 919; CG 1437 ± 381 min), and the total recorded training load via Polar Team-2 software (Table 1).

### Strength Parameters

The 2 × 3 mixed ANOVA revealed a significant main effect of within subjects factor time for LP (*F* = 8.647, *df* = 1, *p* = 0.007, ${\text{η}}_{\text{p}}^{2}$ = 0.265) and for LC (*F* = 5.865, *df* = 1, *p* = 0.023, ${\text{η}}_{\text{p}}^{2}$ = 0.196) but no group ^∗^ time effect was observed. *Post hoc* analysis showed a significant increase in Frel from pre-to-post for the EG in LP (*p* = 0.009) and in LC (*p* = 0.026). Frel remained unchanged for TG and CG in the two test machines (Table 2).

Group comparison showed no differences between the groups at Baseline in LP and LC. At Posttest EG showed a significant higher Frel in LC (*p* = 0.036) compared to CG. No differences were observed between the groups at Posttest in the LP machine.

### Myofiber Diameter

**FIGURE 4:**
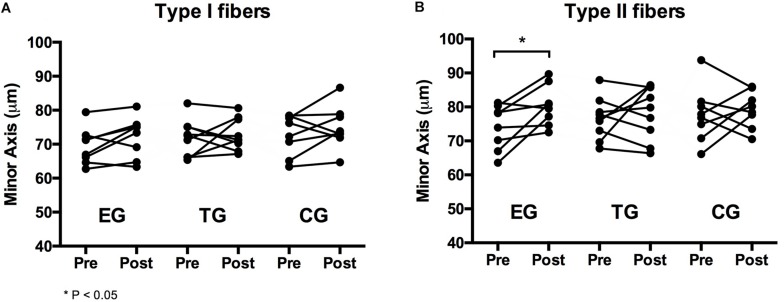
Individual changes in myofiber diameter of **(A)** type I and **(B)** type II fibers in the EMS-Group (EG), Training-Group (TG), and Control-Group (CG) measured 2 weeks before (Pre) and after the intervention period (Post).

For type I fibers no significant time or time ^∗^ group effect was observed. The analysis of the type II fibers showed a significant effect over time (*F* = 4.369, *df* = 1, *p* = 0.048, ${\text{η}}_{\text{p}}^{2}$ = 0.166) but no group ^∗^ time interaction effect. *Post hoc* analysis detected a significant increase in type II myofiber diameter (minor axis) for EG at pre-to-post (*p* = 0.023). Myofiber diameter remained unchanged in TG and CG. One-sided ANOVA for group comparison showed no differences between the groups at Baseline and Posttest for type I and type II (Table 3 and Figure 4).

### MTOR Signaling Proteins

**FIGURE 5:**
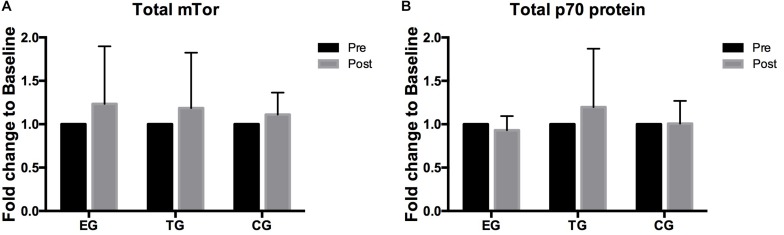
Total levels of **(A)** mTOR, **(B)** p70s6k in EMS-Group (EG), Training-Group (TG), and Control-Group (CG) measured 2 weeks before (Pre) and after the intervention period (Post). Values are presented in means ± *SD*.

A 2 × 3 ANOVA of repeated measures revealed no effects over time and no interaction effect for total mTOR and total p70s6k. No group differences were observed between the groups at Baseline or Posttest (Figure 5).

### Correlations

A significant positive correlation (*r* = 0.355; *n* = 23; *p* = 0.048) was shown between changes (%delta = Posttest−Baseline) in Frel and changes in muscle fiber size of type II fibers. Significant increases in LP Frel are associated with increases in LC Frel (*r* = 0.349; *n* = 26; *p* = 0.040).

## Discussion

The study was designed to investigate the effects of superimposed WB-EMS on muscular adaptations during a competitive season in trained soccer players. After 7 weeks we observed significant increases in myofiber size of 8.9 ± 8.5% in type II fibers only in the EG but not in TG and CG. Although insignificant, EG showed also the highest increase in type I muscle fiber size of 4.1 ± 5.5% compared to TG and CG. Regarding strength capacity we also observed significant increases in Frel at LP (15.1 ± 13.3%) and at LC machine (8.5 ± 9.3%) only in the EG. The results in maximal strength are in line with the result of our previous investigation with professional soccer players (Filipovic et al., 2016) and comparable to the increases shown in local EMS studies with trained and elite athletes after 12–28 sessions (cf. Willoughby and Simpson, 1998; Maffiuletti et al., 2000, 2002a,b; Babault et al., 2007; Billot et al., 2010; Filipovic et al., 2012). It seems that 3 × 10 squat jumps twice per week in addition to two to four soccer training sessions or soccer training alone have no significant effects on muscular adaptation and strength capacity of the leg muscles. Accordingly, we could isolate the effect of the EMS stimulus and address the observed effects to the combination of WB-EMS and soccer training.

We assume that the increase of the myofiber diameter was small because all of the subjects had previous strength training experience and were trained soccer players. It has also to be accounted for that the entire net time under tension in the TG and EG were 28 min (2 × 2 min per week). A regular strength training unit with moderate movement and speed, consisting of three exercises with 5 sets and 10 repetitions for the leg muscles will result in a time under tension of approximately 25 s per set, 6 min for the entire session and around 90 min in 7 weeks. This would represent a more than threefold loading time of muscle. The EG and TG conducted in total only 14 training sessions (approx. 12–15 min) over a 7-week time period. In a comparable EMS study by Gondin et al. (2011) increases of 12% in type I and 23% type II fibers were observed after 8 weeks (three sessions/week) in trained athletes in combination with usual sport-specific training (4–6 h per week). We applied a significant lower number of sessions in total (14 vs. 24 session) and also a significant lower time under tension per week (4 vs. 12.5 min). Compared to regular hypertrophic strength training, WB-EMS provided a minimalistic time pattern of stimulation on muscle fibers. In comparable studies applying resistance exercise on subjects, two to three training sessions a week (approx. 70%; 3–5 sets; 8–12 reps) are associated with increased strength capacities and muscular hypertrophy (Izquierdo et al., 2009; Andersen and Aagaard, 2010).

Electromyostimulation provides a stimulus where during a voluntary recruitment of myofibers; artificially, high supramaximal recruitment of myofibers is generated (Gregory and Bickel, 2005). Fast type II muscle fibers are recruited from the beginning; in addition to, the small type I fibers even at relative low intensities and moderate movement velocities suggest a preferential activation of the type II fibers (Gregory and Bickel, 2005). This offers a high, but short mechanical strain on myofibers implying also a high neuronal activation of motor units at the motoric endplate (cf. Hortobagyi and Maffiuletti, 2011). This probably does not include involvement of supraspinal neuronal centers to a higher extent than voluntary contractions with high force output, therefore explaining only moderate changes in general strength abilities; however, EMS may exert mechanical stimulation of myofibers to a high extent. Although this can be assumed, it will probably not mimic the myofibrillar loading that occurs during regular resistance training. Differences in the activation of signaling proteins between EMS and regular resistance exercise modes are to date not well investigated. High mechanical strain of myofibers is associated with increased signaling via mTOR pathway reflected by increased protein synthesis (Egerman and Glass, 2014; Gehlert et al., 2015b). Due to the time point of biopsy sampling in the rested state, we were unable to detect acute changes in the activation of mTOR-related signaling via phosphorylation of mTORC1 and p70s6k between and within groups and instead determined total levels of p70s6k and mTOR. We did not determine direct changes of these proteins in any of our groups. First, the time course of stimulation might has been be too short and second the accumulation of total protein levels will probably not limit the system in generating substantial hypertrophy; however, the cellular communication via p70s6k will not strictly reflect protein synthesis rates exceeding the acute phase after resistance exercise (Bamman et al., 2018). After 7 weeks we detected significant increased Type II diameter in the EG pointing to a substantial recruitment and stimulation of this fiber type during EMS (cf. Gregory and Bickel, 2005). Only small increases were observed in type I fibers. Type I fibers need a higher time under tension and also have a significantly lower capacity for hypertrophy (Bagley, 2014). We further have to assume that regular soccer training, associated with high energy turnover in muscle, will at least partly inhibit mTOR-related signaling (Coffey and Hawley, 2017) and prevent substantial increases in mTOR and p70s6k protein. The inhibition of growth via concurrent pathway interaction can also explain the lower degree in hypertrophy in the EG due to endurance-based soccer training (Fyfe et al., 2014) especially in type I fibers.

Regarding increase in muscle size of the *m. quadriceps femoris* and its influence on strength capacity of the leg extensor it is likely that the increases in Frel of the LC machine are also associated to an increase in muscle mass of the hamstrings. Regarding injury prevention, this could reduce the risk of hamstring injuries that represent one of the most common injuries in sprint demanding sports like soccer (Ekstrand et al., 2011). Thus, WB-EMS can be of great interest in team sports and soccer especially (Goode et al., 2015).

In general, the determined improvements in myofiber diameter and strength show a substantial interindividual variability reflecting a high individual pattern in the adaptation (high/low/none-responder) to the EMS stimulus. This was observed in previous EMS studies (Kreuzer et al., 2006; Wirtz et al., 2015, 2016; Filipovic et al., 2016; Micke et al., 2018) but also after strength training concerning hypertrophy (Petrella et al., 2008). Furthermore, the differences in playing time, high intensity running, and/or sprint distances during soccer match and training produce great deviations in training/work load within the players (Mohr et al., 2003; Suarez-Arrones et al., 2015; Pettersen et al., 2018) that can influence adaptative processes.

The results reveal that the increase in type II muscle fiber size may have positively influenced the force–velocity characteristic of the whole muscle (cf. Suchomel et al., 2018). However, increases in strength capacity are not only associated with hypertrophy. A variety of structural adaptation mechanism, e.g., muscle fiber shift toward type IIa fibers (Perez et al., 2002; cf. Requena Sanchez et al., 2005) or an increase in muscle stiffness via Ca^2+^-induced modification of titin-isoforms (Rassier, 2017) as well as adaptation in the electromechanical coupling or energetic supply of the muscle (cf. Gehlert et al., 2011; Baird et al., 2012; Gehlert et al., 2015a; Ulbricht et al., 2015) could have been involved to promote increases in strength capacity.

A limitation of the present study was that the weekly training load could not be entirely standardized. This was due to the fact that players, depending on their position, received different tasks during training units and players had also distinct playing time during matches. On the other hand, this was distributed over the entire group of subjects and not only in EG, TG, or CG. Due to extreme difficulties in managing training and the intervention during the training day, it was not possible to collect biopsies acutely after exercise. This would have enabled us to determine acute changes in phosphorylation of mTOR targets and chaperones which might indicate the impact of WB-EMS on fiber recruitment, protein synthesis, and also muscle damage (Jacko et al., 2019). However, the current approach, using well-trained soccer players, created a realistic training situation, which was not scientifically overadapted. Further, we determined that WB-EMS is a safe training method and does not negatively influence players’ performance.

Based on our results we conclude that implemented WB-EMS indeed will not dramatically change muscle adaptive responses when maintaining soccer training during a competitive season. However, it can emphasize the adaptability of fast type II fibers and may therefore result in a fine tuning of strength capacities while maintaining other performance parameters. The results reveal that WB-EMS training can be implemented as a time efficient alternative to traditional strength training in order to increase strength capacity during competitive season in soccer players. It could also be a promising training alternative for other team sports, e.g., track and field, handball, or basketball. More studies should investigate the effects of WB-EMS in other competitive sports in various intensities, training frequencies, and over extended time courses to elucidate chronic and long-term effects of this training tool.

## Data Availability

The datasets for this manuscript will be made available by the authors. Requests to access the datasets should be directed to the corresponding author AF.

## Ethics Statement

This study was carried out in accordance with the recommendations of the “Ethics Committee of the German Sports University Cologne”. All subjects gave written informed consent in accordance with the latest version of the Declaration of Helsinki. The protocol was approved by the “Ethics Committee of the German Sports University Cologne” [06-02-2014].

## Author Contributions

AF, SG, MG, and WB conceived and designed the research. AF conducted the experiments. MD conducted the muscle biopsies. BS assisted the biopsies and supervised the WB-EMS training. AH prepared, processed muscle tissue, and measured the parameters. AF and SG analyzed the data and wrote the manuscript. TS, MD, and WB revised the manuscript. All authors read and approved the manuscript.

## Conflict of Interest Statement

The authors declare that the research was conducted in the absence of any commercial or financial relationships that could be construed as a potential conflict of interest. The handling Editor declared a shared affiliation, though no other collaboration, with several of the authors AF, MG, AH, BS, TS, WB, and SG at the time of the review.
